# Spatial genomics reveals a high number and specific location of B cells in the pancreatic ductal adenocarcinoma microenvironment of long-term survivors

**DOI:** 10.3389/fimmu.2022.995715

**Published:** 2023-01-04

**Authors:** Hosein M. Aziz, Lawlaw Saida, Willem de Koning, Andrew P. Stubbs, Yunlei Li, Kostandinos Sideras, Elena Palacios, Jaime Feliu, Marta Mendiola, Casper H. J. van Eijck, Dana A. M. Mustafa

**Affiliations:** ^1^ Department of Surgery, Erasmus University Medical Center, Rotterdam, Netherlands; ^2^ Department of Pathology & Clinical Bioinformatics, The Tumor Immuno-Pathology Laboratory, Erasmus University Medical Center, Rotterdam, Netherlands; ^3^ Department of Pathology & Clinical Bioinformatics, Erasmus University Medical Center, Rotterdam, Netherlands; ^4^ Divisions of Medical Oncology and Hematology, Mayo Clinic, Rochester, MN, United States; ^5^ Department of Pathology, La Paz University Hospital, IdiPAZ, Madrid, Spain; ^6^ Department of Medical Oncology, La Paz University Hospital, IdiPAZ, Madrid, Spain; ^7^ Cátedra UAM-ANGEM, Madrid, Spain; ^8^ Centro de Investigación Biomédica en red de Cáncer, CIBERONC, Instituto de Salud Carlos III, Madrid, Spain; ^9^ Molecular Pathology and therapeutic Targets Group, La Paz University Hospital, IdiPAZ, Madrid, Spain

**Keywords:** pancreatic ductal adenocarcinoma, long-term survival, B cells, tumor immune microenvironment, gene expression, spatial genomics

## Abstract

**Background and aim:**

Only 10% of pancreatic ductal adenocarcinoma (PDAC) patients survive longer than five years. Factors underlining long-term survivorship in PDAC are not well understood. Therefore, we aimed to identify the key players in the tumor immune microenvironment (TIME) associated with long-term survivorship in PDAC patients.

**Methods:**

The immune-related gene expression profiles of resected PDAC tumors of patients who survived and remained recurrence-free of disease for ≥36 months (long-term survivors, n=10) were compared to patients who had survived ≤6 months (short-term survivors, n=10) due to tumor recurrence. Validation was performed by the spatial protein expression profile of immune cells using the GeoMx™ Digital Spatial Profiler. An independent cohort of samples consisting of 12 long-term survivors and 10 short-term survivors, was used for additional validation. The independent validation was performed by combining qualitative immunohistochemistry and quantitative protein expression profiling.

**Results:**

B cells were found to be significantly increased in the TIME of long-term survivors by gene expression profiling (*p*=0.018). The high tumor infiltration of B cells was confirmed by spatial protein profiling in the discovery and the validation cohorts (*p*=0.002 and *p*=0.01, respectively). The higher number of infiltrated B cells was found mainly in the stromal compartments of PDAC samples and was exclusively found within tumor cells in long-term survivors.

**Conclusion:**

This is the first comprehensive study that connects the immune landscape of gene expression profiles and protein spatial infiltration with the survivorship of PDAC patients. We found a higher number and a specific location of B cells in TIME of long-term survivors which emphasizes the importance of B cells and B cell-based therapy for future personalized immunotherapy in PDAC patients.

## Introduction

Pancreatic ductal adenocarcinoma (PDAC) is a lethal malignancy and it is estimated to become the second leading cause of global cancer-related mortality in the near future (1, 2). Its annual fatality rates worldwide have become nearly comparable to its incidence rates (3). Due to its extremely infiltrative nature and rapid tumor spread, for all stages combined, only 5-10% of the patients survive for 5 years or longer. Even patients with early-stage PDAC undergoing surgery relapse at exceedingly high rates with approximately only 25% surpassing 5-year survival time (1, 3, 4). So far, current and emerging treatment strategies have been poorly effective in prolonging survival for these patients (1). Based on the accumulating insights into the importance of the immune system for the outcome of pancreatic cancer patients, understanding alterations in the immune landscape of PDAC is essential to establish new personalized immunotherapeutic approaches to combat this devastating disease (5).

Tumor cells and their surrounding microenvironment (TME) are closely related and interact constantly (6). The TME of PDAC consists of fibroblasts, immune cells, pancreatic stellate cells (PaSCs), adipocytes, and extracellular matrix (ECM) (7). These cells and structures collectively create desmoplasia in PDAC (8), which is present in both primary tumors and metastatic lesions, and compose more than 50% of PDAC tissue (9). The extremely dense fibrotic desmoplasia prevents immune cell infiltration and vascularization, thus limiting exposure to conventional systemic therapy (10–12). One of the main components of desmoplasia is mesenchymal originating cells (cancer-associated fibroblasts (CAFs)). CAFs secrete ECM proteins like alpha-smooth muscle actin (αSMA), fibronectin, and various types of collagens (13). In addition, desmoplasia consists of endothelial cells and epithelial cells that carry fibroblastic features such as the expression of fibroblast-specific protein 1 (FSP-1) (14). The desmoplasia in PDAC functions as the traffic that organizes the penetration of immune cells into tumor areas (15). Moreover, it plays a crucial role in tumorigenesis, and it is one of the reasons why immunotherapy thus far has not met its promise in PDAC compared to a variety of other malignancies (16, 17). In addition, myofibroblast depletion has been linked to favorably altering the composition of the immune infiltrate in the TME of PDAC stroma (18). Therefore, further characterization of critical components of the TME could provide a better understanding and add guidance to overcome therapeutic resistance in PDAC.

The resilience of pancreatic cancer towards currently available therapeutics is due to various reasons including the ability of cancerous cells to alter the immune system during disease progression (5). As PDAC develops, it creates a favorable tumor immune microenvironment (TIME) that supports the structure of cancer instead of attacking it (19). The TIME of PDAC is characterized by high infiltration and activation of immunosuppressor cells such as myeloid-derived suppressor cells (MDSC), that orchestrate multiple signaling pathways to stimulate cancer progression, angiogenesis, and metastasis (20). The MDSCs inhibit the antitumor immunity *via* various mechanisms including the crosstalk with other immunosuppressive cell types such as regulatory T cells (Tregs), M2 differentiated tumor-associated macrophages (TAMs) and T helper 2 (Th2) differentiated CD4^+^ T cells (5, 21–24). Tregs infiltration in PDAC tissue is correlated with advanced and progressed disease (25) and furthermore with poor outcome (26). They protect tumor cells from attacking immune cells by the secretion of IL10, and TGF-Beta (25). In addition, the balance between inhibitory receptors (CTLA-4, PD1, BTLA, LAG-3, CD40L) and the co-stimulatory molecules for T cell function (CD28, OX40, GITR, CD137, CD27) on the surface of T cells is disturbed in PDAC (27). Therefore, the immune effector cells like cytotoxic CD8^+^ T and natural killer (NK) cells, dendritic cells (DC), T helper 1 (Th1) T cells, and M1 differentiated macrophages that promote anti-tumor activity seem inactive in PDAC (5, 23, 28).

In a previous study, we described several key changes including deactivation of immune effector cells and mobilization of the immunosuppressors in the TIME of pancreatic cancer (5). However, PDAC is not only characterized by a profound local, but also by systemic tumor immune suppression associated with early disease recurrence and poor survival (29). Outlining the critical components and differences within the TIME in tissue samples of resected long-term compared to short-term survivors of PDAC using gene expression and digital spatial profiling has not been reported before. Investigating the main immune-related cellular and molecular differences between these two groups will guide future unique immunotherapeutic approaches in PDAC patients.

## Methods

### Patients and clinical data

We retrospectively assessed fresh frozen (FF) tumor tissue samples from patients who underwent resection for histologically proven PDAC between December 2004 and December 2016, at the Erasmus MC University Medical Center (EMC) in Rotterdam, the Netherlands. Patients were screened for eligibility based on their survival time. We selected treatment naïve tumors of patients following surgical resection for their PDAC that remained recurrence-free and survived for at least 3 years (36 months, long-term survivors). We compared these tumors to those of patients who survived less than 6 months (short-term survivors) due to tumor recurrence. We excluded patients who died from postoperative complications and other causes. Furthermore, patients with neuroendocrine, duodenal, distal-bile duct, and ampullary carcinoma were also excluded.

Clinical, histopathological, and laboratory data were retrieved from the electronic medical records. Information obtained from pathology reports included: tumor grade (well, moderate, or poor), lymph node status (positive and negative), tumor location (head, body, or tail), tumor stage (according to the AJCC 8^th^ edition), and margin status (radical (R0) vs non-radical (R1; ¾ 1mm)). From the laboratory data, we collected baseline cancer antigen (CA) 19-9 (kU/L), carcinoembryonic antigen (CEA) (ng/ml) and, calculated the systemic immune inflammation index (SIII) (29). The study was approved by the Medical Ethical Committee of EMC (MEC-2020-0252).

Intergroup differences in baseline characteristics (short-term vs. long-term survivors) on continuous variables were determined using the non-parametric Mann-Whitney *U* test, and categorical data were compared using Fisher’s exact test. Cancer-specific survival and recurrence-free survival were calculated from the date of surgery to the date of the event (death from cancer or recurrence of cancer, respectively). In the case of no event, the information of the patients was censored at the date of the last follow-up. Patients were followed according to the standard of care guidelines (30). Follow-up information was retrieved through the electronic medical records and by contacting patients’ general practitioners in the event of missing information. Significance for statistical tests was inferred at a *p*-value of <0.05. All statistical analyses, if not mentioned otherwise, were performed using SPSS (version 26.0).

**Figure 1:**
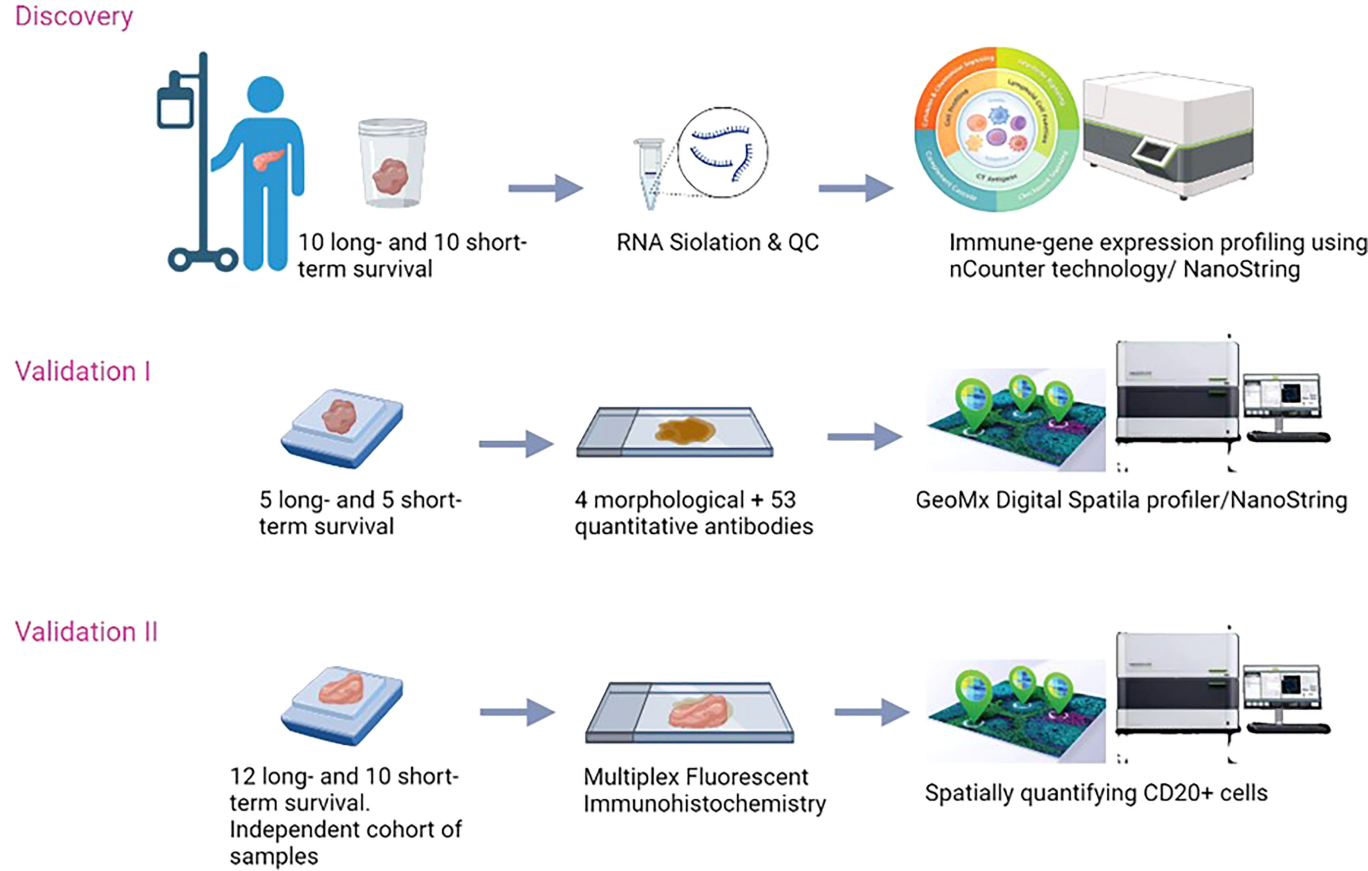
Schematic overview of the study.

To validate our results, an independent cohort of samples was collected from La Paz University hospital of Madrid. Formalin-Fixed, Paraffin-Embedded (FFPE) samples of patients who had the same characteristics as the discovery cohort, were selected. The study was approved by the Medical Ethical Committee of the University of Madrid (PI3468). The overview of the study is presented in (**Figure 1**).

### RNA samples

Fresh Frozen (FF) tumor samples of the included PDAC patients were sectioned with 5 µM thickness, stained with hematoxylin and eosin (H&E), and examined by a pathologist who highlighted the location of the tumor-rich areas. Each sample was then further sectioned, and a total of 60 µM was used for RNA isolation using the Fresh Frozen Micro Kit (QIAGEN, Hilden, Germany) according to the manufacturer’s protocol. The quality and quantity of RNA samples were examined using the Agilent 2100 Bioanalyzer (Agilent Technologies, Santa Clara, California, USA). RNA concentration was corrected to include the proportion of the sample that was >300 base pairs.

### Targeted gene expression profiling of the RNA samples

To measure the differences in the immune infiltration, the nCounter^®^ PanCancer Immune Profiling Panel (NanoString Technologies, Seattle, WA) was used. This panel consisted of 730 cancer immune-related genes, 40 housekeeping genes, 6 positive and 8 negative controls. RNA samples of 200 ng/5-7 uL were hybridized to target probes for 17 hours at 65°C. RNA expression levels were measured using the nCounter^®^ FLEX Instrument (31). The counting of the genes was done by scanning 490 Fields Of Views (FOV).

### Data analysis of the targeted gene expression

Gene count data were analyzed using the nSolver™ Analysis Software (version 4.0) and its nSolver™ Advanced Analysis module (version 2.0) (www.nanostring.com). The quality control of the measurements was done according to the general workflow used in the nSolver™ Analysis Software (32). The expression levels of the negative controls were used to determine the detection limit by calculating the average of the negative control expression plus double the standard deviation. The expression data were normalized using the most stable housekeeping genes using the geNorm algorithm (33). The differentially expressed genes were identified using the recommended workflow of the nSolver™ Advanced Analysis, which includes a mixture of negative binomial models, simplified negative binomial models, or log-linear models based on the convergence of each gene. Genes were regarded differentially expressed if the *p*-value was < 0.05 and the |Fold-Of-Change (FOC)| > 2.

### Cell type profiling

To identify the immune cell types, candidate marker genes that define the cell types were tested for their specificity and stable expression, following our previously described method (34). The assumption for appropriate marker genes is that all selected genes for the same cell type decrease and increase in the same direction. This was tested by calculating the correlation of determination (*R^2^*) and the slope for each pair of genes within a cell type definition. The gene pairs with a correlation of determination between 0.4 and 0.6 were checked separately using scatter plots to calculate their slope. The slope should be above 0.75 and below 1.25 which is equivalent to being between 33.75 and 56.25 degrees to indicate high pairwise similarity (35) between the candidate genes and to be included in the analysis (**Supplementary Table 1**). Genes that did not pass the above criteria were excluded from cell type profiling analysis by changing the Probe Annotation and cell types contrast matrix in nSolver™ Advanced Analysis. Independent two-sample *t*-tests were conducted between the short-term and long-term survivors using the cell type scores calculated in the cell type profiling analysis.

### Validation by using the GeoMx™ digital spatial profiling

FFPE samples of the same PDAC samples used in the targeted gene expression profile were used for validation in the GeoMx™ Digital Spatial Profiling analysis. A total of 10 samples were retrieved (5 samples from long-term and 5 samples from the short-term survivors). From each sample, one section of 5µM was used to be stained with GeoMx Solid Tumor TME Morphology Kit Human Protein Compatible (NanoString Item # 121300301) that contains: Pan-Cytokeratin (PanCK, 647 nm), alpha-smooth muscle actin (αSMA, 488 nm), CD45 (594 nm). Nuclear stain CYTO-13 (NanoString Item # 121300303) was used to detect all cells (532 nm). At the same time, the sections were incubated with a cocktail of 53 photo-cleavable, oligo-labeled primary antibodies (the Onco-Immune protein panel: 4-1BB, αSMA, B7H3, Bcl2, beta-2-microglobulin, CD11c, CD20, CD127, CD14, CD163, CD25, CD27, CD3, CD34, CD4, CD40, CD45, CD45RO, CD56, CD66b, CD68, CD8, CD80, CD86, CTLA4, EpCAM, ER-alpha, FAPa, FoxP3, GITR, GZMB, fibronectin, Her2/ErbB2, Ox40L, HLA-DR, ICOS, IDO1, Ki-67, LAG3, MART-1, NY-ESO-1, OX40-L, Pan-Cytokeratin, PD-1, PD-L1, PD-L2, PR, PTEN, S100b, STING/TMEM173, TIM-3, and VISTA). The incubation with antibodies was done overnight according to the manufacturer’s protocol. Afterward, slides were loaded onto the DSP instrument. Each slide was first scanned to produce a digital image of tissue morphology based on the fluorescent markers. Next, the regions of interest (ROI) were selected which was guided by the morphological markers. Finally, the photocleaved oligos from the spatially resolved ROIs were hybridized to the corresponding probes for 17 hours at 65°C and scanned using 280 fields of views (FOV). Microplates were analyzed using DSP data center software (www.nanostring.com).

### Selection of ROIs

Multiple ROIs were selected based on morphological markers including tumor-rich ROIs with a high morphological expression of PanCK, desmoplastic with a high morphological expression of αSMA, and immune-rich containing a high expression of CD45. However, some ROIs contained more than one feature and were therefore relabeled/classified as “tumor plus desmoplasia” or “desmoplasia plus CD45” ROIs. In addition, some ROIs contained all features, so they were categorized as “tumor plus desmoplasia plus CD45” (**Supplementary Figure 1**). DNA staining was used to ensure selecting ROIs that contained cells. The size and the number of cells included in selected ROIs can vary.

### Data analysis of the GeoMx™ DSP

The protein expression data generated by the GeoMx™ DSP were first normalized for technical variation during hybridization by using the positive controls included in the experiment (The External RNA Control Consortium (ERCC)). Subsequently, a normalization based on housekeeping genes that showed a stable expression across all samples (Histone 3 and S6 Ribosomal Protein) was conducted. GAPDH, a third housekeeping gene, was excluded from being used in normalization because it was not stably expressed in all ROIs and all samples (**Supplementary Figure 1G**). These two subsequent normalizations enables comparing ROIs of different size and number of cells to each other. The first comparison was performed between ROIs that contained tumor (i.e. tumor, tumor plus desmoplasia, and tumor plus desmoplasia plus CD45) and ROIs that contained stroma (i.e. desmoplasia, desmoplasia plus CD45, and CD45) in long-, and short-survivors separately (**Supplementary Figure 1** and **Supplementary Table 2**). The non-parametric Mann-Whitney *U* test was used to determine differentially expressed proteins. After that, a correlation analysis using Spearman’s rank-order correlation was run for all the significant proteins in the tumor-rich and stromal ROIs aiming to identify immune cell types that infiltrate together in various areas of PDAC tissue samples based on the survival of the patients. The correlations were regarded to be positively significant when the correlation coefficient rho (r) > 0.5, and negatively significant when r < -0.5 (36). Finally, to identify the cell types that were infiltrated with B cells, a correlation analysis was performed on all detected proteins in ROIs that showed high CD20 expression.

### Validation using an independent cohort of samples

A total of 22 FFPE samples were used to confirm our results (n=12 long-term survival, n=10 short-term survival). Samples were sectioned (5µm thickness) and stained with Pan-Cytokeratin antibody that is available in the GeoMx Solid Tumor TME Morphology Kit Human Protein Compatible (NanoString Item # 121300301) (PanCK, 647 nm), Nuclear stain CYTO-13 (NanoString Item # 121300303) was used to detect all cells (532 nm). Anti-CD20 antibody (Abcam Recombinant Anti-CD20 antibody [EP459Y] (ab78237) was labeled with antibody labeling kit from Invitrogen Thermo Fisher called Alexa Fluor™ 594 Antibody Labeling Kit Item number A20185 (594 nm). Sectioned samples were hybridized with the immune cell profiling panel, consisting of 20 antibodies (PD-1, CD68, HLA-DR, Ki-67, Beta-2-microglobulin, CD11c, CD20, CD3, CD4, CD45, CD56, CD8, CTLA4, GZMB, PD-L1, PanCk, SMA, Fibronectin). The panel contains three positive controls (Ribosomal protein S6, Histone 3, and GAPDH) and three negative controls (two mouse and one rabbits IgGs) that were used in each measurement. Samples were prepared according to the manufacturer’s protocol (NanoString, GeoMx DSP, Seattle, USA). The GeoMx DSP instrument was used to perform the measurements and the software (v2.2).

## Results

### Clinicopathological characteristics

A total of twenty samples of PDAC patients were included in the discovery cohort, 10 in the long-term survival group, and 10 in the short-term survival group. Median cancer-specific survival was 53.1 and 4.7 months, respectively, whereas the median time-to-recurrence was 46.3 and 3.7 months, respectively. The main characteristics of the patients were matched to the best of our ability. There were no significant differences in age, gender, tumor location, operation procedure, tumor differentiation, lymph node status (positive vs. negative), margin status (R0 vs. R1), T-stage (T1-T2 vs. T3), adjuvant systemic therapy, SIII, CEA, or CA19-9. Baseline clinicopathologic characteristics are summarized in **Supplementary Table 3**.

In addition, a total of twenty-two samples of PDAC patients were included in the validation cohorts, 12 in the long-term survival group, and 10 in the short-term survival group. Also for these samples, the main characteristics of the patients were matched to the best of our ability. The clinicopathologic characteristics of the independent validation cohort were similar, except for T-stage, to the discovery cohort of samples and were summarized in **Supplementary Table 4**. However, when comparing the short-term survival group to the short-term survival group, and the long-term survival group to the long-term survival in the discovery cohort and the validation cohort, respectively, tumor grade was found to be significantly different in the short-term survival groups.

### Thirty-nine immune-related genes were differentially expressed

**Figure 2:**
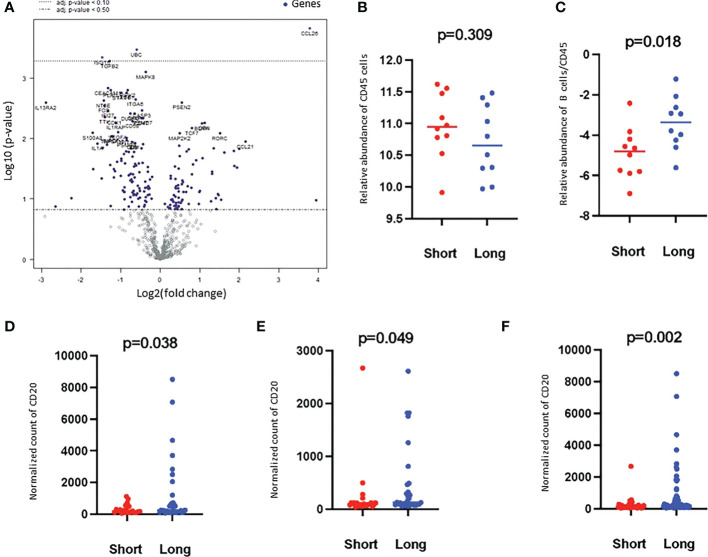
Volcano plot of the differentially expressed genes and Higher B cell scores were found in long-term survivors. **(A)** Volcano plot of the differentially expressed genes in the long- compared to short-term survivors of PDAC. Each dot indicates a detected gene. Three genes were differentially expressed after multiple corrections: CCL26 was overexpressed, and UBC and ISTG were downregulated in long-term survivors. The dotted line represents *p*-value < 0.1. **(B)** The relative abundance of CD45 cells in long and short-term survival samples (Independent two-sample *t*-tests). Each dot presents a sample, the line presents the average expression in each group, y-axis presents the relative expression of CD45 which indicates no significant differences. **(C)** The score of B cells relative to the total infiltration of CD45+ was found to be significantly higher in the long-term survival group (Independent two-sample *t*-tests). Each dot presents a sample, the line presents the average expression of genes identify B cells relative to CD45 in each group, y-axis present the relative expression of B cells/CD45 which indicates a higher abundance of B cells in the long-term survival group. The protein counts of B cell (CD20+) were confirmed to be highly expressed in long-term survivors using the GeoMx™ DSP technology. **(D)**. in the stroma, **(E)**. in between tumor-rich areas and **(F)**. in desmoplastic and tumor-rich areas combined analysis (non-parametric Mann-Whitney *U* test). Each dot presents an ROI, the y-axis is the normalized counts of CD20 antibody. Blue color presents long- and red color present short-term survival samples.

Of the 730 immune-related genes, 631 genes were above the detection threshold (i.e. 21 counts), and 99 of these genes were found to be differentially expressed between the two groups (short-term vs. long-term survivors) with a *p*-value < 0.05. Thirty-nine genes had |FOC|>2, among which 14 genes were over-expressed, and 25 genes were under-expressed in long-term survivors compared to short-term survivors (**Supplementary Table 5**; **Figure 2A**). Four of the over-expressed genes in long-term survivors were related to the function of B cells, namely, CR2, CD19, CD79B, and BLNK (**Supplementary Table 5**). Only three genes were found to be differentially expressed genes after multiple corrections, therefore, we presented the differences between long- and short-term survivals based on immune cell types.

### Higher B cell infiltration was found in long-term survivors

Based on the cell type profiling, the total infiltration of immune cells (*PTPRC* (CD45^+^)) was comparable between long- and short-term survivors (*p*=0.309, **Figure 2B**). A significantly higher B cell score (CD19, CD22, CR2, and *MS4A1* (CD20)) was found in long-term survivors compared to short-term survivors (*p*=0.018, **Figure 2C**). None of the other cell types were found to be significantly different between the two groups (**Supplementary Figure 2**). The higher infiltration of B cells in long-term survivors was confirmed by using the GeoMx™ DSP analysis using the FFPE samples corresponding to the discovery cohort (**Table 1**). B cells were found to be significantly higher in the stroma of the long-term survival samples (*p*=0.038, **Figure 2D**). They also were found to be higher within tumor cells of the same group (*p*=0.049, **Figure 2E**). The comprehensive analysis that included the desmoplastic and the tumor-rich area showed a significantly higher infiltration of B cells in the long-term survival group of samples (*p*=0.002, **Figure 2F**).

**Table 1:** Comparison of tumor ROIs in the short-term (23) vs. long-term (30) survival group.

PROTEINS	MEAN SHORT	STD. DEVIATION	MEAN LONG	STD. DEVIATION	P-VALUE
**CD20**	234.12	540.80	**467.81**	676.73	0.049399
**CD3**	1041.42	1011.72	**2041.61**	1966.48	0.030576
**CD34**	1109.32	1220.56	**1737.00**	1099.81	0.011393
**CD4**	735.96	446.13	**1444.55**	1284.17	0.035756
**CD8**	1286.85	1793.04	**2754.67**	2106.55	0.001537
**EPCAM**	4814.94	3509.35	**7280.00**	4231.73	0.022658
**HLADR**	3854.88	7675.85	**4382.50**	2965.26	0.024880
**PAN CYTOKERATIN**	**33471.73**	25602.47	21493.35	16178.91	0.040771
**B41BB**	**45.33**	16.69	38.76	28.02	0.025452
**B7H3**	**2140.52**	843.31	1263.33	668.10	0.000499
**BETA2MICROGLUBLIN**	**877.97**	371.10	697.93	319.16	0.021613
**CD127**	**956.44**	533.72	421.64	162.43	0.000011
**CD25**	**178.19**	52.02	124.19	48.79	0.000320
**CD66B**	**687.04**	1012.77	314.50	524.17	0.022655
**CD80**	**92.16**	44.22	57.90	36.27	0.002495
**CTLA4**	**738.22**	552.79	171.89	94.21	2.1538E-8
**FAPA**	**516.50**	362.36	154.84	137.64	0.000014
**FOXP3**	**69.41**	40.26	33.64	14.19	0.000088
**GITR**	**49.95**	22.21	33.61	14.85	0.005557
**KI67**	**4068.24**	4943.67	1205.49	1284.28	0.000234
**LAG3**	**31.61**	12.74	22.99	8.11	0.011983
**GAPDH**	**32276.460870**	15699.606503	13176.806667	6668.252984	7.3035E-7
**LIVERARGINASE**	**207.62**	178.43	108.81	82.07	0.002422
**OX40L**	**201.87**	128.64	78.84	34.37	0.000003
**PD1**	**115.88**	22.01	86.68	30.74	0.001491
**PDL1**	**63.36**	16.15	44.15	16.94	0.000218

Higher mean values (absolute counts) indicate higher infiltration in comparison to the other group.

The bold values present the up-regulated proteins in a given group.

The first 7 proteins were up-regulated in long-term survival group ad the rest were up-regulated in short-term survival group.

### B cells infiltrated within tumor cells close to T cells in long-term survivors

Tumor ROIs that expressed high levels of CD20 in long-term survival showed a high expression of various T cell markers like CD3, CD4 and CD8 and of HLADR marker that might be expressed by memory B cells or dendritic cells (DCs) or antigen-presenting cells (APCs) (**Supplementary Table 6**). Moreover, the expression of CD20 was associated with the high expression of CD34 which is a bone marrow stem cell marker that is expressed by progenitor cells of blood vessels and stromal tissue and may reflect the increased number and quality of blood vessel formation in long-term survivors (37, 38). Furthermore, a strong correlation between the expressions of CD20 and CD27 (r= 0.752, p<0.001) and Bcl2 (r= 0.734, p<0.001) was found in tumor areas of long-term survivors, which may reflect a specific subtype of B cells like memory B cells. All proteins which were correlated with CD20 expression are summarized in **Supplementary Table 6**; **Supplementary Figures 3**, **4**.

### Sensitivity analysis of the CD20-rich ROIs

Since we found higher infiltration of CD20-positive cells in the tumor tissue of the long-term survival group compared to the short-term survival group, and high correlations were found between CD20-positive cells and CD3, CD4, and CD8-positive cells, we examined high CD20-positive ROIs between short and long-term survival groups. We found that the expression of CD20 correlates negatively with the expression of αSMA (r=-0.656, *p*=0.002) and with fibronectin (r=-0.540, *p*=0.014) in long-term survivors. Positive correlations were found between CD20 and Bcl2 (r=0.695, *p*=0.001), CD3 (r=0.598, *p*=0.550), CD4 (r=0.550, *p*=0.012), CD8 (r=0.567, *p*=0.009) and Ki67 (r=0.558, *p*=0.011), which confirm our previous findings in the tumor areas.

### Homogeneous infiltration of suppressive immune cells was found in tumor and desmoplastic areas of short-term survivors

**Figure 3:**
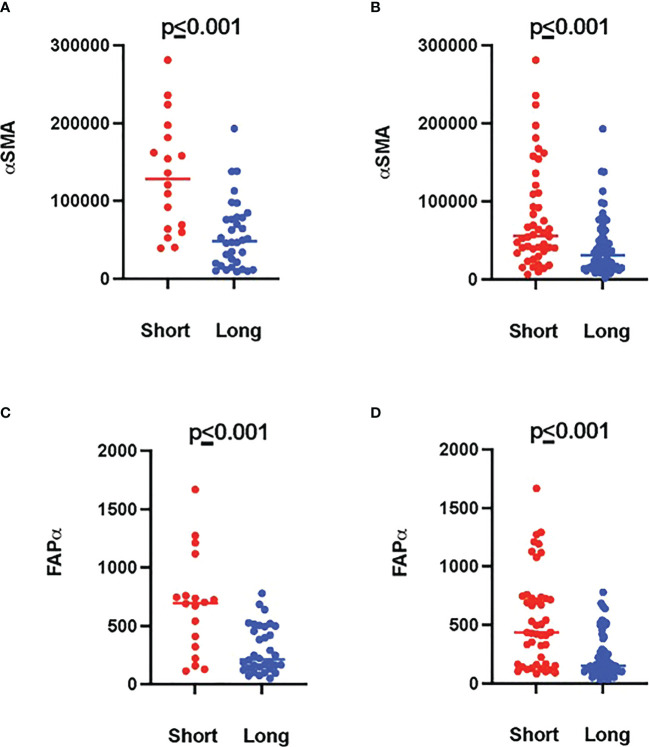
Higher amounts of desmoplastic proteins were found in short-term survivors. **(A)** The expression of αSMA in the desmoplastic areas, **(B)** in the desmoplastic and tumor-rich areas combined. Y-axis is the normalized counts of αSMA antibody. **(C)** The expression of FAPa proteins in the desmoplastic areas, **(D)** in the desmoplastic and tumor-rich areas combined. Y-axis is the normalized counts of FAPa antibody. Each dot presents an ROI and the line presents the average normalized counts of the antibody in each group. Blue color presents long- and red color presents short-term survival samples. The two proteins were found to be significantly higher in short- compared to long-term survivors using the GeoMx™ DSP technology (non-parametric Mann-Whitney *U* test).

Tumor ROIs of short-term survivors revealed a high expression of molecules correlated with immune suppression such as PD-1, PD-L1, CTLA4, LAG3, FoxP3, and CD25 compared to long-term survivors (**Table 1**). The same suppressive molecules were found to be highly expressed in desmoplastic areas (**Table 2**). In addition, the level of αSMA and FAPa expression was significantly higher in desmoplastic areas of short-term survivors compared to long-term survivors (*p*=0.001, **Table 2**; **Figures 3A, C**). Importantly, our results do not show differences in the percentage of the desmoplastic areas between the two groups, it shows differences in the desmoplastic protein expression levels in very similar sizes of desmoplastic ROIs in short- and long-term survivors. The comprehensive analysis that included the desmoplastic and the tumor-rich area showed the same results (**Figures 3B, D**). All differentially expressed and correlated proteins in tumor and stromal areas are summarized in **Supplementary Tables 6**, **7**.

**Table 2:** Comparison of stromal ROI’s in the short-term (18) vs. Long-term (34) survival group.

PROTEIN	MEAN SHORT	STD. DEVIATION	MEAN LONG	STD. DEVIATION	P-VALUE
**CD20**	219.87	144.22	1171.37	2016.96	0.037773
**αSMA**	132117.36	72586.74	56723.61	43634.38	0.000222
**B41BB**	62.72	15.69	51.24	19.55	0.035187
**B7H3**	2539.79	911.22	1857.21	1105.65	0.016204
**CD11c**	2679.86	2562.20	4693.02	4895.68	0.039579
**CD127**	578.43	177.90	408.20	202.25	0.000618
**CD14**	623.22	347.11	1112.55	690.59	0.000876
**CD163**	521.70	428.57	985.02	675.14	0.001318
**CD25**	177.37	33.63	138.71	47.84	0.001505
**CD3**	1881.55	1342.51	3322.39	2993.45	0.014575
**CD34**	1391.17	1025.02	2154.74	1529.17	0.025669
**CD4**	1238.74	714.35	2127.06	1427.09	0.005950
**CD56**	442.22	218.69	385.16	336.98	0.036034
**CD68**	4258.12	4154.54	9157.21	11880.80	0.022086
**CD8**	2140.40	1992.50	4602.11	3717.32	0.000876
**CD80**	119.94	45.17	76.24	34.81	0.001505
**CTLA 4**	2530.39	2018.74	357.77	322.27	0.000004
**ERalpha**	75.37	35.62	97.04	32.26	0.012403
**FAPα**	677.83	429.86	297.34	200.09	0.000711
**FoxP3**	79.35	42.15	36.20	15.15	0.000033
**GAPDH**	24542.27	8249.41	10663.87	7331.43	0.000002
**HER2ErbB2**	176.04	95.49	108.37	38.96	0.000190
**HLADR**	3267.98	2025.63	5074.52	2835.21	0.013093
**KI67**	1360.91	1213.17	893.62	1326.22	0.008411
**LAG3**	52.21	36.87	37.17	18.83	0.019433
**LiverArginase**	267.44	279.97	146.90	88.72	0.032753
**OX40L**	348.33	243.02	168.22	175.31	0.000575
**PD1**	158.78	38.46	107.58	34.10	0.000054
**PDL1**	83.91	22.01	58.39	27.63	0.000197
**S100b**	736.12	715.40	1366.93	1367.72	0.017077
**VISTA**	399.12	266.00	224.01	103.47	0.012398

Higher mean values (absolute counts) indicate higher infiltration in comparison to the other group.

Interestingly, we found a strong negative correlation between αSMA and CD20-positive cells (r=-0.749, *p*<0.001, **Supplementary Table 7**) in the stromal ROI of the short-term survivors. Negative correlations were also found between αSMA and other immune effector cells such as CD8-positive cells (r=-0.505, *p*=0.014, **Supplementary Table 7**), suggesting that these effector immune cells are unable to penetrate the stroma of short-term survivors. On the other hand, immunosuppressive cells, and receptors such as FoxP3, CTLA4, and PD-L1 were all positively associated with αSMA. Analyzing CD20-rich areas, we found similar results in long-term survivors. That is, CD20 was negatively associated with αSMA (r=-0.762, *p*=0.028), CTLA4 (r=-0.762, *p*=0.028), Fibronectin (r=-0.738, *p*=0.037), and GZMB (r=-0.810, *p*=0.015).

## Validation of our findings

**Figure 4:**
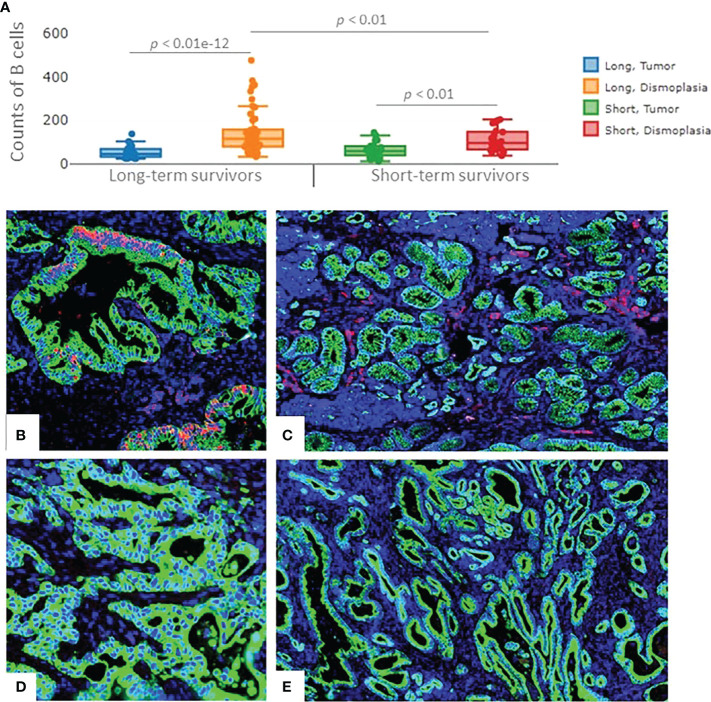
The expression of B cells in the validation cohort of samples. **(A)** Significant higher infiltration of B cells was found in the desmoplastic areas of the long-term survivors, compared to desmoplastic areas of short-term survivors. Infiltration of B cells was found to be higher in desmoplasia compared to tumor-rich areas in both groups. Each dot represents an ROI, the middle line presents the average of the normalized expression of CD20 antibody, long-term survivors n=12, short-term survivors n=10. Fluorescent immunohistochemistry staining of PDAC samples (Mixed Linear Model test). **(B)** CD20 cells (B cells) infiltrated in-between (within) the tumor cells, **(C)** and in the desmoplastic areas of long-term survival PDAC samples. **(D)** negative staining of CD20 in-between (within) tumor cells, **(E)** Fewer B cells were found to infiltrate in the desmoplastic areas of short-term survival PDAC samples. Green color: Pan Cytokeratin, Red color: CD20, blue color: nucleus.

A higher number of B cells in long-term survivors was validated using the independent cohort of PDAC samples (*p=*0.01, **Figure 4A**). B cells were found to be exclusively infiltrated in between tumor cells in the long-term survival group. The infiltration of B cells in the validation samples was higher in the desmoplasia area as compared to the tumor-rich areas. Fluorescent immunohistochemistry of CD20 (B cell marker) confirmed that B cells infiltrated in desmoplastic areas and in between tumor cells in some samples of the long-term survival group (**Figures 4B–E**). Of note, we did not find differences in the tertiary lymphoid structures between the two groups.

## Discussion

**Figure 5:**
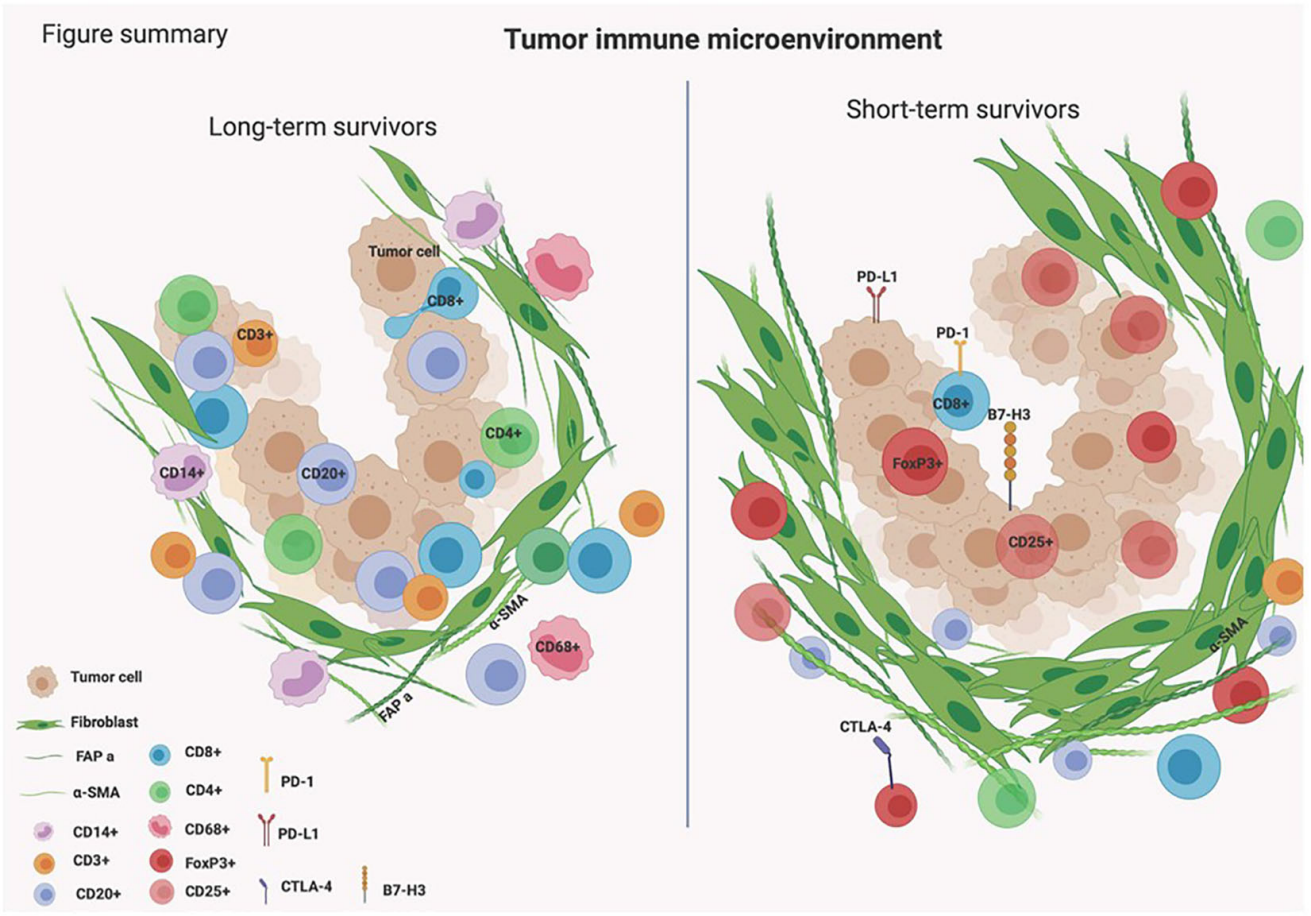
Schematic presentation of the TIME in long- and short-term survivors of PDAC patients.

Unveiling the complex biology of the TIME in PDAC is essential for finding novel effective immunotherapeutic targets. This study highlights the presence of B cells infiltrating the TIME in tumors of PDAC patients who survived longer than 3 years. B cells were found to be infiltrated in between tumor cells and we hypothesize that they might be orchestrating essential immune responses causing the infiltration of other types of immune cells within the tumors of long-term survivors of PDAC (**Figure 5**).

The role of B cells in PDAC is not completely understood (39, 40). There is growing evidence to consider B cells as key regulators of the tumor-induced immune response (41). B cells have been shown to be an essential part of tumor-infiltrating lymphocytes (TILs) infiltration in PDAC (42). The scattering infiltration of B cells or their organized presence in tertiary lymphoid structures in PDAC has been associated with improved survival (43). It has also been shown that pancreatic cancer infiltrating B cells can recognize mutant KRAS epitopes and can produce antibodies against KRAS presenting tumor cells (44). Our results indicated that B cells were found close to T cells in long-term survivors which might suggest that CD20^+^ cells might be acting as APCs. In addition, the associated subset of T cells in long-term survivors displayed an activated effector phenotype (high associations to HLA-DR and CD45RO). These results were achieved when using the GeoMx DSP technique that allowed studying the differences in immune infiltration in a specific location. By using bulk RNA, that was used in gene expression profile, the differences of T cells and APCs between long- and short-term survivors were not clear. Taken together, our results highlight the added value and importance of spatial biology techniques that enable understanding the immune regulation at a high level of details. In the same line of our results, it was shown before that long-term survival present a unique list of neoantigens which is associated with a weaker immunosuppressive environment (more CD8+ T cells, MUC16- and CA125) (45). The data from the gene expression analysis showed that four of the over-expressed genes in long-term survivors were associated with “B cell function”, and none in short-term survivors. The over-expressed gene *CD79B* in long-term survivors was previously shown to function as a heterodimer involved in signal transduction *via* the BCR (46). The presence of CD20^+^ cells and CD8^+^ cells and their co-localization was shown to improve antitumor response and was correlated with increased survival in ovarian cancer, breast cancer, and malignant melanoma (47–49). These data support the previous finding that the presence of B cells within the TIME of PDAC is associated with a favorable prognosis (50). Moreover, they are active representatives of the TIME in pancreatic cancer alongside T cells, making them prime candidates for future (pre) clinical research. To dissect the exact role of B cells within the TIME of PDAC, the subtypes of B cells such as regulatory B cells, plasma B cells, and memory cells need to be further characterized (50, 51).

Another interesting finding of this study was the high expression of αSMA in short-term survivors. The changes in the composition of ECM in short-term survivors might be the main barrier that prevents TILs to infiltrate near tumor cells (**Figures 3**, **5**), making them immunologically ‘cold’ tumors. While high-quality neoantigens are present in PDAC cells (45), they appear to be immune-edited over time only in long-term survivors (52). Also, the CAFs and the ECM composition of desmoplastic regions continuously change as cancer develops (53). There are several types of CAFs in PDAC including myofibroblastic, inflammatory, and antigen-presenting fibroblasts (15). The cancer-derived Transforming Growth Factor-β (TGF-β) in PDAC patients plays an important role in converting normal fibroblasts into myofibroblastic CAFs (54), which produce αSMA, and fibroblast activation protein (FAP) (55). Our data show that these two proteins are strongly expressed within the stromal ROIs in short- compared to long-term survivors (**Tables 1**, **2**; **Figure 3**). Moreover, the expression of αSMA and FAP was negatively associated with the infiltration of B- and T cells in short-term survivors. Therefore, we think that targeting αSMA and FAP CAFs in combination B cell therapy might be a good option. TGF-β gene expression was 2.4 times higher in short-term survivors than in long-term survivors. Taken together, the data of this study hypothesized that the high expression of TGF-β might be one of the main drivers that induces CAFs to produce a high quantity of αSMA and FAP that prevent TILs to infiltrate tumor areas. It is important to highlight that we did not study the percentage of desmoplastic areas or the tumor stromal density (TSD) in PDAC tissue samples, like it was performed previously (56). We rather quantified the desmoplastic-related protein expression in ROIs of similar sizes in short- and long-term survival. High TSD was shown to be associated with lower metastasis and favorable outcome in resectable patients.

Results of this study suggest that treating PDAC can perhaps be achieved by targeting TGF-β, to reduce the production of αSMA, in combination with B cell therapy. B cell therapy remains in its infancy especially in the case of PDAC since its role has been debatable (43, 57). However, recent studies have shown adopting certain culture conditions or gene insertion, costimulatory ligands have been forced into the expression of B cells in order to increase their immunostimulatory activation capacity. Also, tumor-specific antigen presentation by ex vivo antigen loading or by engineering B cells with a B cell receptor specific to tumor antigens has been improved recently (58, 59). With the recent expansion of the knowledge of B cells in cancer, new therapeutic options are anticipated to grow in the upcoming years. Previously, targeting stroma in PDAC has been disappointing (19), however, recent studies showed that CAFs depletion (using nab-paclitaxel or FAP specific therapies) is potentially a positive treatment approach. In addition, reprogramming CAFs into quiescent fibroblasts (using all-trans retinoic acid (ATRA, STARPAC trial) or vitamin D) has shown promising results in treating PDAC (15, 60). Nevertheless, none of these pre-clinical trials showed complete remission of disease in these patients. Combining B cell therapy with reprogramming or CAFs depletion might improve the survival of PDAC patients.

Despite our effort of adding an external validation cohort, the sample size of the total cohort is one of the major limitations of our study, which is mainly due to the scarcity of long-term survivors of PDAC. This could also explain why no major differences were found in the clinicopathological characteristics of short-term and long-term survival groups. Furthermore, we found a difference between tumor grade in the short-term survivors in the discovery and validation cohort. However, we believe that this has to do with tumors that were classified as moderate to poor. Namely, in the discovery cohort, we called these tumors poorly differentiated tumors. When not using this classification, there were no differences between the two cohorts.

## Conclusions

Our study underlines the importance of B cell infiltration in tumors of PDAC patients. B cells infiltrated in higher quantities in stromal areas and were found exclusively intra-epithelial in long-term survivals of our cohort of samples. The spatial data revealed that the large presence of B cells was associated with the infiltration of T cells and APCs. In contrast, not only B cells but also the diversity of immune cells was much lower in short-term survivors. In the TME of short-term survivors, the high expression of αSMA was associated with low diversity and infiltration of immune cells to tumor areas. Our study hypothesized potential roads ahead for revolutionizing PDAC combination treatments with focus on B cells, targeting stromal reactions, and anti-TGF-β molecules.

## Data availability statement

The data presented in the study are deposited in the GEO repository, accessionnumber GSE220929.

## Ethics statement

The studies involving human participants were reviewed and approved by Medical Ethical Committee of EMC (MEC-2020-0252) Medical Ethical Committee of the University of Madrid (PI3468). The patients/participants provided their written informed consent to participate in this study.

## Author contributions

HA, LS, DM performed the measurements and the analysis research. HA, LS, WK, AS, YL and DM integrated and analyzed the data. HA, WK, YL and DM created the Figures. EP, JF and MM provided the validation cohort of samples. DM, CE conceived and supervised the project. All authors contributed to the article and approved the submitted version.

## Supplementary material

Supplementary Figure 1 Examples of the various ROIs that were selected in PDAC samples for the GeoMx™ DSP Legends: **(A)** An ROI positive for Pan-Cytokeratin, presenting a tumor area. **(B)** An ROI positive for αSMA, presenting a desmoplastic area. **(C)** An ROI positive for CD45, presenting an immune-rich area. **(D)** An ROI positive for Pan-Cytokeratin and αSMA, presenting an area that combined tumor and desmoplasia. **(E)** An ROI positive for αSMA and CD45, presenting an immune-rich desmoplastic area. **(F)** An ROI positive for Pan-Cytokeratin, αSMA, and CD45, presenting an immune-rich tumor and desmoplastic area. Yellow = Pan-Cytokeratin, green = αSMA, red = CD45+ cells, blue = nucleus DNA. The size of the ROI and the number of cells included in each ROI varies between the different selection. The 2-step normalization (data analysis) enables comparison between ROIs of different sizes. Long exposure settings for the morphological markers were used to capture low levels of expression and ensure accurate ROI selection. The intensity of the morphological markers does not affect the protein expression of the quantified antibias. The morphological markers are used to guide the selection of ROIs only. **(G)** The expression of the three housekeeping genes in all ROIs in PDAC samples that were measured with the GeoMx™ DSP technology.

Supplementary Figure 2 Scores of various types of immune cells in long- and short-term survivors. Legend: none of the identified immune cell types were found to be significantly scored between the two groups.

Supplementary Figure 3 Heat-maps of the significant proteins correlated with the expression of CD20 cells in long- compared to short-term survivors. Legend: **(A)** heat-map of the significant proteins that show correlation with CD20 in tumor ROIs in long- and short-term survivors. The expression of CD20 (B cells) is correlated with CD3, CD4, and CD8 (T cells) ad with HLA-DR (antigen presenting cells) in tumor areas of long-term survivors. The expression of CD20 is negatively correlating with CD127, CD25, and FoxP3 (regulatory T cells) and many other immune checkpoint and regulatory proteins. **(B)** heat-map of the significant proteins that show correlation with CD20 in stromal ROIs in long- and short-term survivors. The expression of CD20 (B cells) is also correlated with CD3, CD4 and CD8 (T cells) and with HLA-DR (antigen presenting cells) and with CD14, CD163 (monocytes and myeloid cells) in stromal areas of long-term survivors.

Supplementary Figure 4 Dot plots of the most significant proteins correlated with CD20 expression in long term survivors. Legend: **(A)** Dot plots of the significantly highly expressed proteins in tumor ROIs in long-term survivors. **(B)** Dot plots of the significantly highly expressed proteins in stromal ROIs in long-term survivors. Each dot presents an ROI, the middle line presents the average counts of antibodies, y-axis presents the normalized counts of the specific antibody.
