# Supplementary figures and images for: Spatial genomics reveals a high number and specific location of B cells in the pancreatic ductal adenocarcinoma microenvironment of long-term survivors
Source: Front Immunol. 2023 Jan 4;13:995715. doi: 10.3389/fimmu.2022.995715 (PMC9846531; doi:10.3389/fimmu.2022.995715)

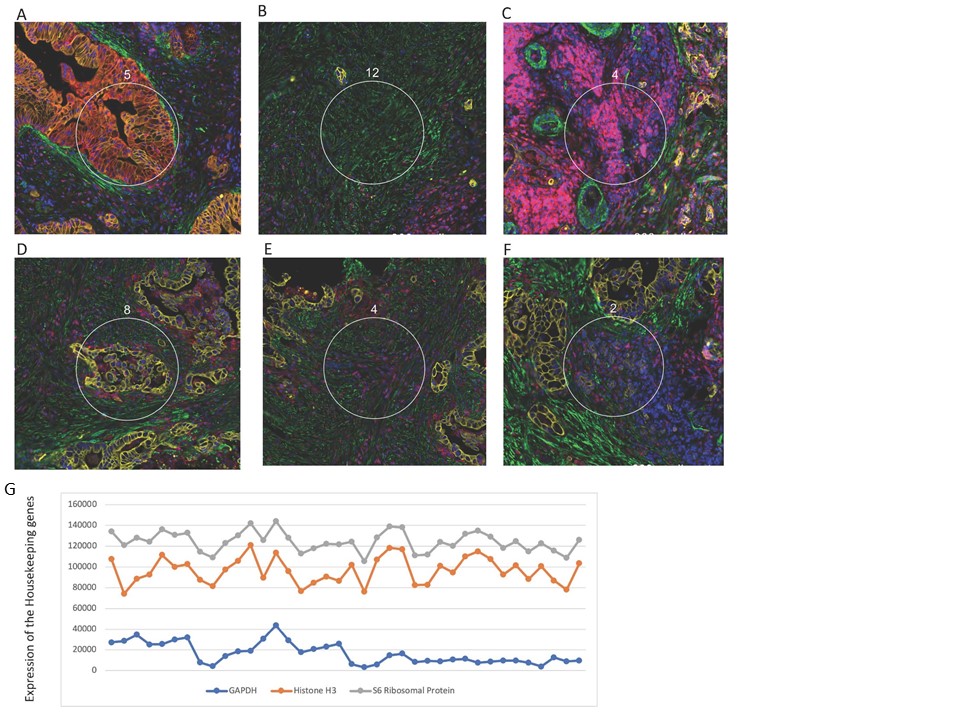

Supplement: Supplementary Figure 1 — Examples of the various ROIs that were selected in PDAC samples for the GeoMx™ DSP Legends: (A) An ROI positive for Pan-Cytokeratin, presenting a tumor area. (B) An ROI positive for αSMA, presenting a desmoplastic area. (C) An ROI positive for CD45, presenting an immune-rich area. (D) An ROI positive for Pan-Cytokeratin and αSMA, presenting an area that combined tumor and desmoplasia. (E) An ROI positive for αSMA and CD45, presenting an immune-rich desmoplastic area. (F) An ROI positive for Pan-Cytokeratin, αSMA, and CD45, presenting an immune-rich tumor and desmoplastic area. Yellow = Pan-Cytokeratin, green = αSMA, red = CD45+ cells, blue = nucleus DNA. The size of the ROI and the number of cells included in each ROI varies between the different selection. The 2-step normalization (data analysis) enables comparison between ROIs of different sizes. Long exposure settings for the morphological markers were used to capture low levels of expression and ensure accurate ROI selection. The intensity of the morphological markers does not affect the protein expression of the quantified antibias. The morphological markers are used to guide the selection of ROIs only. (G) The expression of the three housekeeping genes in all ROIs in PDAC samples that were measured with the GeoMx™ DSP technology. [file Image_1.jpeg]

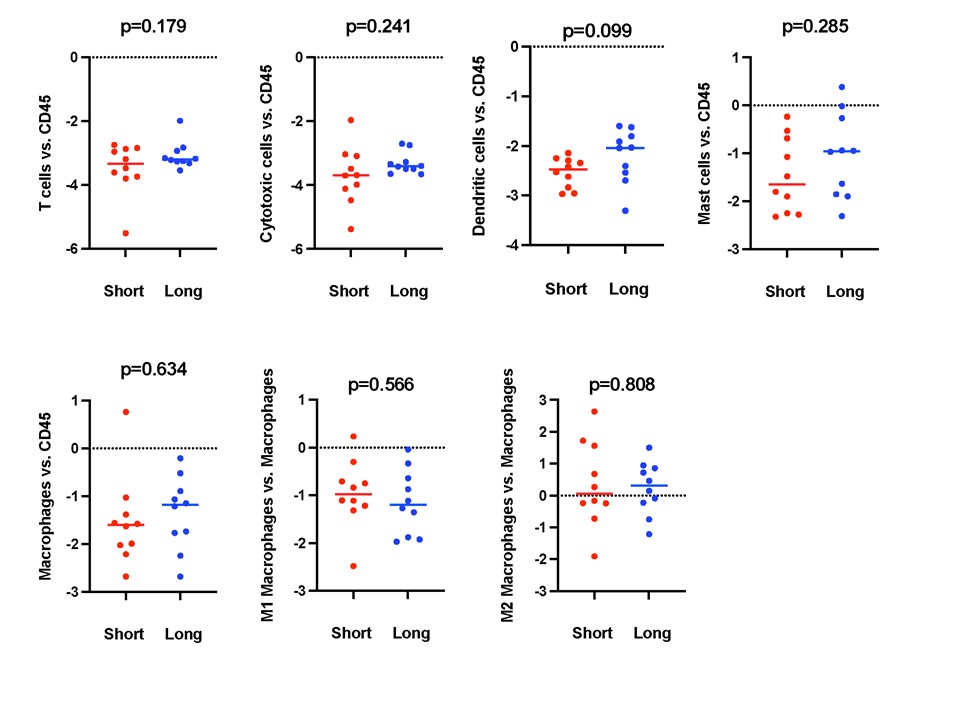

Supplement: Supplementary Figure 2 — Scores of various types of immune cells in long- and short-term survivors. Legend: none of the identified immune cell types were found to be significantly scored between the two groups. [file Image_2.jpeg]

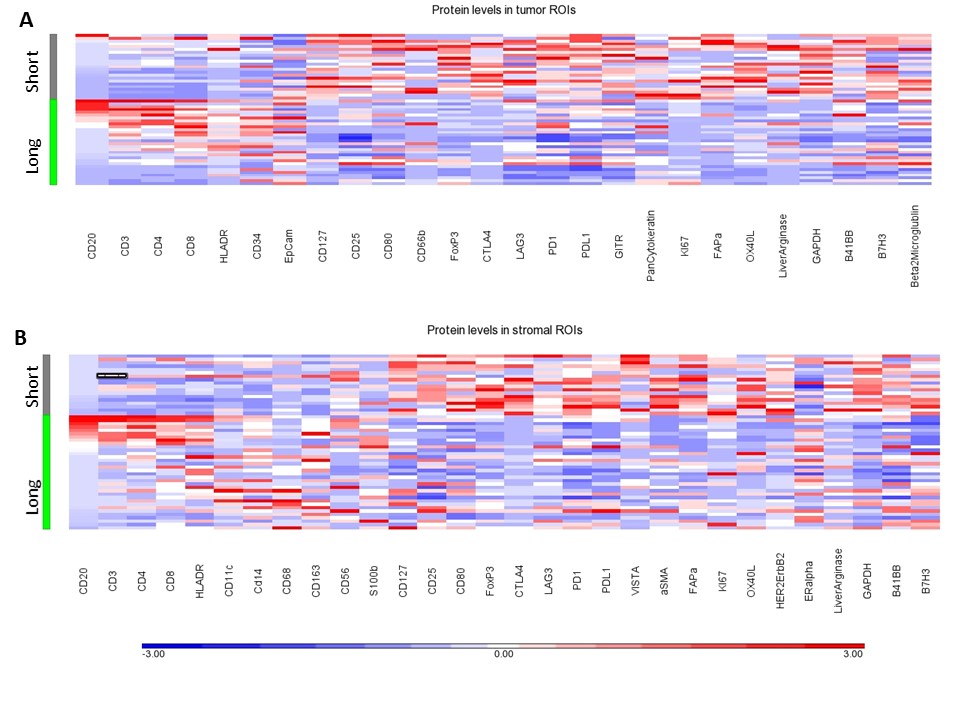

Supplement: Supplementary Figure 3 — Heat-maps of the significant proteins correlated with the expression of CD20 cells in long- compared to short-term survivors. Legend: (A) heat-map of the significant proteins that show correlation with CD20 in tumor ROIs in long- and short-term survivors. The expression of CD20 (B cells) is correlated with CD3, CD4, and CD8 (T cells) ad with HLA-DR (antigen presenting cells) in tumor areas of long-term survivors. The expression of CD20 is negatively correlating with CD127, CD25, and FoxP3 (regulatory T cells) and many other immune checkpoint and regulatory proteins. (B) heat-map of the significant proteins that show correlation with CD20 in stromal ROIs in long- and short-term survivors. The expression of CD20 (B cells) is also correlated with CD3, CD4 and CD8 (T cells) and with HLA-DR (antigen presenting cells) and with CD14, CD163 (monocytes and myeloid cells) in stromal areas of long-term survivors. [file Image_3.jpeg]

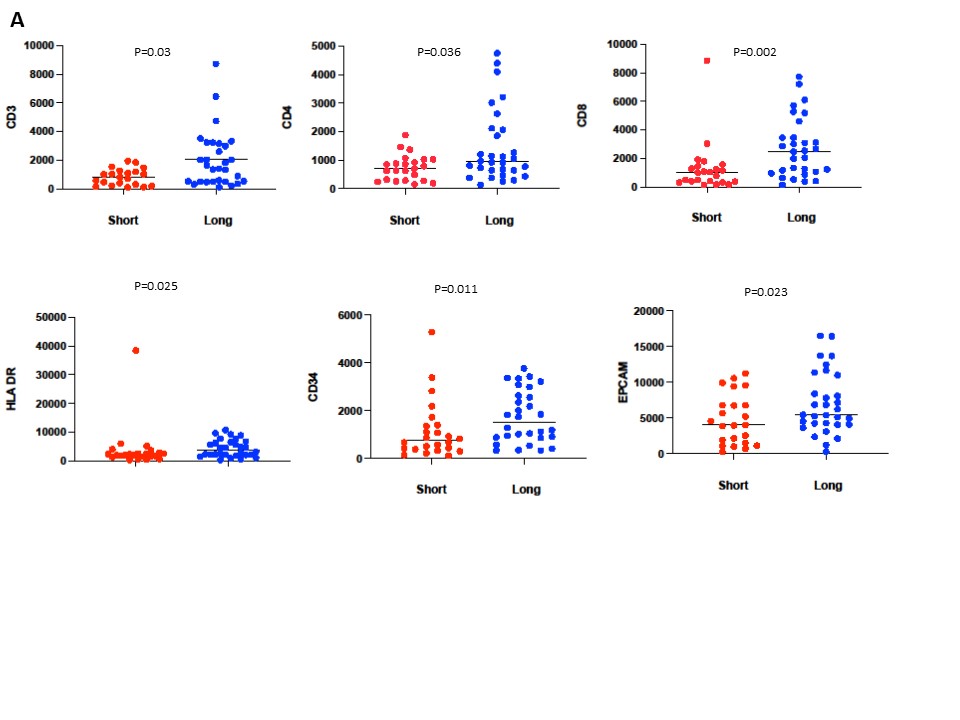

Supplement: Supplementary Figure 4 — Dot plots of the most significant proteins correlated with CD20 expression in long term survivors. Legend: (A) Dot plots of the significantly highly expressed proteins in tumor ROIs in long-term survivors. (B) Dot plots of the significantly highly expressed proteins in stromal ROIs in long-term survivors. Each dot presents an ROI, the middle line presents the average counts of antibodies, y-axis presents the normalized counts of the specific antibody. [file Image_4.jpeg]

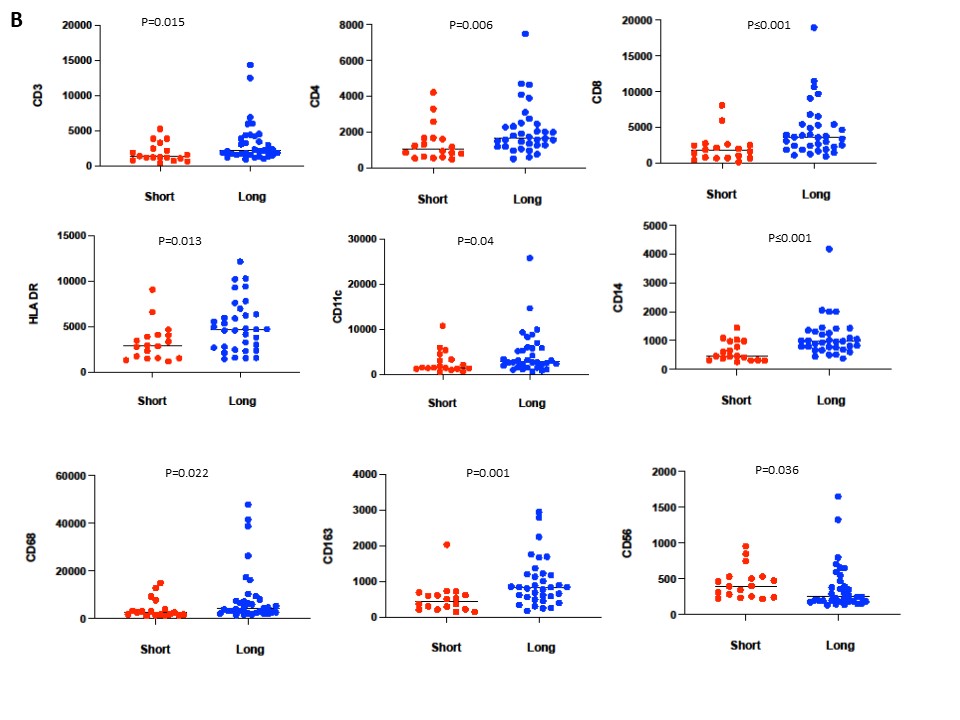

Supplement: Supplementary file 5 [file Image_5.jpeg]
