# Supplementary material for: Spatial genomics reveals a high number and specific location of B cells in the pancreatic ductal adenocarcinoma microenvironment of long-term survivors
Source: Front Immunol. 2023 Jan 4;13:995715. doi: 10.3389/fimmu.2022.995715 (PMC9846531; doi:10.3389/fimmu.2022.995715)
Supplement: Supplementary file 6 [file Table_1.docx]

**Additional Supplementary File 1**

**Supplementary Table 1:** Cell type profiling, selection of the representative genes per cell type

| Cell type: representative gene subset | Genes | Correlation Coefficient | Slope | Include |
| --- | --- | --- | --- | --- |
| B cells: *CD19, CD22, CR2, MS4A1* | *CD19-CD22*  *CD19-CR2*  *CD19-MS4A1*  *CD22-CR2*  *CD22-MS4A1*  *CR2-MS4A1* | 0.863  0.953  0.953  0.825  0.898  0.929 | 0.88  0.63  0.65  0.53  0.61  0.97 | Yes  Yes  Yes  Yes  Yes  Yes |
| T cells: *SH2D1A, CD6, CD3G, CD3E, CD3D* | *SH2D1A-CD6*  *SH2D1A-CD3G*  *SH2D1A-CD3E*  *SH2D1A-CD3D*  *CD6-CD3G*  *CD6-CD3E*  *CD6-CD3D*  *CD3G-CD3E*  *CD3G-CD3D*  *CD3E-CD3D* | 0.614  0.738  0.678  0.822  0.655  0.82  0.716  0.823  0.777  0.792 | 0.79  0.7  0.63  0.88  0.48  0.6  0.6  0.81  0.88  0.91 | Yes  Yes  Yes  Yes  Yes  Yes  Yes  Yes  Yes  Yes |
| Regulatory T cells: *FOXP3* | *FOXP3-IL2RA* | -0.052 |  | No |
| CD4 Memory T cells: *CD4* | *CD4-Shell* | 0.138 |  | No |
| CD45: *PTPRC* | *PTPRC* |  |  | Yes |
| CD8 T cell: *CD8A, CD8B* | *CD8A-CD8B* | 0.557 | 0.68 | Yes |
| Exhausted T cells: LAG3 | *LAG3-TIGIT* | 0.457 | 0.55 | No |
| Conventional Dendritic cells 2: *ITGAM, ITGAX* | *ITGAM-ITGAX* | 0.455 | 0.49 | No |
| M2 macrophages: *CD631, MRC1* | *CD163-MRC1* | 0.78 | 0.99 | Yes |
| M1 macrophages: *CD86, CD80* | *CD86-CD80* | 0.436 | 0.49 | No |
| MACROPHAGES: *CD68, FCGR2A* | *CD68-FCGR2A* | 0.733 | 0.82 | Yes |
| Dendritic cells: *CD1A, CD1C* | *CD1A-CD1C* | 0.541 | 0.56 | No |
| Mast CELLS: MS4A2, TPSAB1 | *MS4A2-TPSAB1* | 0.913 | 0.78 | Yes |
| Monocytes: *CD14* | *CD14-CD33*  *CD14-TLR2*  *CD33-TLR2* | 0.203  0.294  0.342 |  | No  No  No |
| Neutrophils: *CSF3R* | *FCGR3A-CSF3R* | -0.001 |  | No |
| totoxic cells: *GZMA, GZMB, PRF1, GZMH* | *GZMA-GZMB*  *GZMA-GZMH*  *GZMA-KLRB1*  *GZMA-KLRD1*  *GZMA-KLRK1*  *GZMA-PRF1*  *GZMB-GZMH*  *GZMB-KLRB1*  *GZMB-KLRD1*  *GZMB-KLRK1*  *GZMB-PRF1*  *GZMH-KLRB1*  *GZMH-KLRD1*  *GZMH-KLRK1*  *GZMH-PRF1*  *KLRB1-KLRD1*  *KLRB1-KLRK1*  *KLRB1-PRF1*  *KLRD1-KLRK1*  *KLRD1-PRF1*  *KLRK1-PRF*1 | 0.42  0.85  0.535  0.387  0.871  0.711  0.628  -0.212  0.393  0.239  0.752  0.14  0.458  0.634  0.831  0.128  0.642  0.189  0.246  0.484  0.595 | 0.31  0.89  0.5  0.79  0.74  0.91  1.08  0.81  0.55  0.83  0.62  0.27  0.68 | No  Yes  No  No  Yes  Yes  Yes  No  No  No  Yes  No  Yes  No  Yes  No  No  No  No  No  No |

**Supplementary Table 2**

Summary of all ROIs that were included in the data analysis of the GeoMx DSP of the first discovery cohort

| **ROIs in long** | **Number in long** | **Number in short** | **Category in data analysis** |
| --- | --- | --- | --- |
| Tumor | 3 | 5 | Tumor Plus |
| Tumor + desmoplastic | 3 | 4 |  |
| Tumor + desmoplastic + CD45 | 24 | 20 |  |
| desmoplastic | 3 | 5 | Stroma Plus |
| Desmoplastic + CD45 | 24 | 23 |  |
| CD45 | 7 | 7 |  |

**Supplementary Table** **3:** Baseline clinicopathological characteristics of the discovery cohort of samples.

| *KOLOM1* | VARIABLES | SHORT-TERM SURVIVAL | LONG-TERM SURVIVAL | P-VALUE |
| --- | --- | --- | --- | --- |
| AGE AT SURGERY, MEDIAN (RANGE) | Years | 64.09 (43.41) | 70.5 (29.63) | 0.541 |
| GENDER | Female  Male | 7  3 | 6  4 | 1.000 |
| TUMOR LOCATION | Head  Body/Tail | 9  1 | 6  4 | 0.303 |
| OPERATION PROCEDURE | Whipple  Distal- and total pancreatectomy | 9  1 | 7  3 | 0.582 |
| TUMOR DIFFERENTIATION | Good  Moderate  Poor | 0  3  7 | 2  6  2 | 0.070** |
| LYMPH NODE METASTASIS | Yes  No | 9  1 | 4  6 | 0.057 |
| MARGIN STATUS | R0  R1 | 5  5 | 5  5 | 1.00 |
| T-STAGE* | T1/T2  T3 | 1  9 | 1  9 | 1.00 |
| SIII | ≥ 900  < 900 | 2  4 | 1  6 | 0.559 |
| Adjuvant systemic therapy*** | Yes (%) | 3 | 5 | 0.650 |
| CEA (ng/ml) | Mean | 7.49 | 2.99 | 0.624 |
|  |  |  |  |  |
|  |  |  |  |  |
| CA19-9 (ku/L) | Mean | 251.0 | 731.78 | 0.744 |
|  |  |  |  |  |

*T-stage; according to the AJCC 8th edition

**P-value indicates Fisher's Exact Test comparing Good and moderate combined vs. Poorly differentiated tumors.

*** Adjuvant therapy consisted of 6 cycles of gemcitabine chemotherapy. Majority of the patients completed treatment while the rest discontinued adjuvant therapy due to toxicity (1 patient in long-term and 1 patient in short-term survival group).

The majority of the patients presented with recurrence at multiple sites; liver and lung metastases accounted for all of recurrences.

**Supplementary Table 4:** Baseline clinicopathological characteristics of the validation cohort of samples.

| *KOLOM1* | VARIABLES | SHORT-TERM SURVIVAL | LONG-TERM SURVIVAL | P-VALUE |
| --- | --- | --- | --- | --- |
| AGE AT SURGERY, MEDIAN (RANGE) | Years | 69.8 (56.80) | 69.1 (52.80) | 0.595 |
| GENDER | Female  Male | 4  6 | 6  6 | 0.485 |
| TUMOR LOCATION | Head  Body/Tail | 8  2 | 9  3 | 0.528 |
| OPERATION PROCEDURE | Whipple  Distal- and total pancreatectomy | 7  3 | 8  4 | 0.645 |
| TUMOR DIFFERENTIATION | Good  Moderate  Poor | 0  9  1 | 1  10  1 | 0.864 |
| LYMPH NODE METASTASIS | Yes  No | 7  3 | 9  3 | 0.583 |
| MARGIN STATUS | R0  R1 | 5  5 | 9  3 | 0.231 |
| T-STAGE* | T1/T2  T3 | 0  10 | 6  6 | 0.032 |
| SIII | ≥ 900  < 900 | 4  6 | 2  10 | 0.675 |
| Adjuvant systemic therapy | Yes  No | 7  3 | 10  2 | 0.45 |
| CEA (ng/ml) | Mean | 3.70 | 4.16 | 0.711 |
|  |  |  |  |  |
|  |  |  |  |  |
| CA19-9 (ku/L) | Mean | 954.0 | 971,91 | 0.520 |

**Supplementary Table 5:** Differentially expressed genes; positive values for FC indicate overexpression of the genes in the long-term survival group, and negative values indicate under-expression of the genes in the long-term survival group.

| Genes | Log2 FC | SE (log2) | Lower CL (log2) | Upper CL (log2) | Linear FC | P-value | Gene.sets |
| --- | --- | --- | --- | --- | --- | --- | --- |
| *CCL26* | 3.78 | 0.792 | 2.23 | 5.33 | 13.7 | 0.000152 | Chemokines |
| *CCL21* | 2.16 | 0.764 | 0.658 | 3.65 | 4.46 | 0.0113 | Chemokines. Regulation |
| *LTF* | 2 | 0.744 | 0.543 | 3.46 | 4 | 0.015 | Humoral immunity |
| *CR2* | 1.94 | 0.826 | 0.324 | 3.56 | 3.85 | 0.0303 | B-Cell Functions |
| *CCL19* | 1.88 | 0.789 | 0.331 | 3.43 | 3.68 | 0.0286 | Chemokines. Regulation |
| *IL22RA* | 1.87 | 0.706 | 0.489 | 3.26 | 3.66 | 0.0162 | Chemokines |
| *TNFRSF13C* | 1.62 | 0.613 | 0.421 | 2.82 | 3.08 | 0.017 | Regulation. TNF Superfamily |
| *RORC* | 1.52 | 0.511 | 0.516 | 2.52 | 2.86 | 0.0082 | Cell Functions |
| *CD36* | 1.35 | 0.499 | 0.372 | 2.33 | 2.55 | 0.0145 | Transporter Functions |
| *CD19* | 1.31 | 0.578 | 0.18 | 2.45 | 2.49 | 0.0356 | B-Cell Functions. Regulation |
| *CD79B* | 1.31 | 0.586 | 0.16 | 2.46 | 2.48 | 0.0385 | B-Cell Functions |
| *SPN* | 1.13 | 0.36 | 0.427 | 1.84 | 2.19 | 0.00556 | Regulation |
| *CXCL2* | 1.07 | 0.425 | 0.24 | 1.9 | 2.1 | 0.0212 | Chemokines. Regulation |
| *BLNK* | 1.06 | 0.337 | 0.399 | 1.72 | 2.08 | 0.00562 | B-Cell functions |
| *VEGFA* | -1.06 | 0.355 | -1.75 | -0.364 | -2.08 | 0.00791 | Cytokines. Leukocyte Functions |
| *ITGA5* | -1.09 | 0.443 | -1.96 | -0.22 | -2.13 | 0.0244 | Adhesion |
| *IL1RAP* | -1.11 | 0.351 | -1.79 | -0.418 | -2.15 | 0.0055 | Cytokine. Regulation |
| *CDK1* | -1.14 | 0.351 | -1.82 | -0.448 | -2.20 | 0.00458 | Cell cycle regulation |
| *CEACAM6* | -1.14 | 0.501 | -2.13 | -0.164 | -2.21 | 0.0345 | Adhesion |
| *IL8* | -1.17 | 0.505 | -2.16 | -0.183 | -2.25 | 0.0322 | Chemokines. Cytokines. Interleukins. Pathogen Defense. Regulation |
| *CXCL6* | -1.17 | 0.541 | -2.23 | -0.112 | -2.25 | 0.0438 | Chemokines. Regulation |
| *TNFSF10* | -1.19 | 0.408 | -1.99 | -0.389 | -2.28 | 0.00926 | Cell Cycle. Cytokines. Regulation. TNF Superfamily |
| *BIRC5* | -1.23 | 0.421 | -2.06 | -0.404 | -2.35 | 0.00918 | Cell Cycle |
| *PLAU* | -1.25 | 0.337 | -1.91 | -0.59 | -2.38 | 0.0016 | Senescence |
| *HLA-C* | -1.26 | 0.526 | -2.3 | -0.232 | -2.40 | 0.0273 | Antigen Processing. Cytotoxicity. Regulation |
| *TGFB2* | -1.27 | 0.301 | -1.86 | -0.676 | -2.40 | 0.000528 | Interleukins |
| *CEACAM1* | -1.31 | 0.351 | -2 | -0.627 | -2.49 | 0.00148 | Adhesion |
| *IFI27* | -1.32 | 0.394 | -2.1 | -0.552 | -2.51 | 0.00349 | Chemokines |
| *TNFRSF11B* | -1.35 | 0.487 | -2.3 | -0.392 | -2.54 | 0.0128 | TNF Superfamily |
| *TTK* | -1.4 | 0.429 | -2.24 | -0.554 | -2.63 | 0.00443 | CT Antigen |
| *FOS* | -1.41 | 0.409 | -2.21 | -0.609 | -2.66 | 0.00286 | Cell proliferation, TGF-beta activation |
| *NT5E* | -1.43 | 0.406 | -2.23 | -0.638 | -2.70 | 0.00237 | Transporter Functions |
| *TREM1* | -1.45 | 0.533 | -2.49 | -0.402 | -2.72 | 0.0143 | Myeloid cells |
| *ISG15* | -1.47 | 0.344 | -2.15 | -0.797 | -2.78 | 0.000457 | Interferons |
| *SAA1* | -1.48 | 0.692 | -2.84 | -0.125 | -2.79 | 0.0462 | Inflammation |
| *IL1A* | -1.58 | 0.561 | -2.68 | -0.477 | -2.99 | 0.0121 | Cytokines. Interleukins |
| *FOXJ1* | -1.69 | 0.722 | -3.1 | -0.27 | -3.22 | 0.0322 | Immune cell suppressor, B cell response inhibitor |
| *S100A8* | -1.71 | 0.576 | -2.84 | -0.586 | -3.28 | 0.00806 | Cell cycle progression, differentiation |
| *IL13RA2* | -2.89 | 0.816 | -4.49 | -1.28 | -7.41 | 0.00255 | Chemokines. T-Cell Functions |

Abbreviations: FC; Fold of change. CL; Confidence Limit. SE; standard error

**Supplementary Table 6.** Correlation analysis in GeoMx™ DSP comparing protein profiles of tumor ROIs of short-term and long-term survivors

| Cell type | Correlated cell type | Correlation coefficient (r) | p-value |
| --- | --- | --- | --- |
| Tumor like ROI’s of the long-term survivors | | | |
| CD20 | CD45  CD45RO  Bcl2  CD27  CD3  CD4  CD44  CD8  ICOS  PR | 0.565  0.649  0.734  0.752  0.825  0.677  0.509  0.674  0.518  0.510 | 0.001  <0.001  <0.001  <0.001  <0.001  <0.001  0.004  <0.001  0.003  0.004 |
| CD3 | CD45  CD45RO  Bcl2  CD11c  CD14  CD20  CD27  CD4  CD44  CD8  FAPa  HLA DR  ICOS  IDO1  PR  S100b Sting  Tmem173  VISTA | 0.707  0.810  0.826  0.518  0.524  0.825  0.798  0.874  0.636  0.813  0.576  0.586  0.712  0.664  0.532  0.500  0.665  0.590 | <0.001  <0.001  <0.001  0.003  0.001  p<0.001  <0.001  <0.001  <0.001  <0.001  0.001  0.001  <0.001  <0.001  0.003  0.005  0.001  0.001 |
| CD4 | CD45  CD45RO  B4-1BB  Bcl2  Beta2Microglublin  CD11c  CD14  CD20  CD25  CD27  CD3  CD40  CD44  CD68  CD8  FAPa  GITR  HLA DR  ICOS  IDO1  LAG3  PDL1  StingTmem173  TIM3  VISTA | 0.851  0.939  0.604  0.713  0.501  0.721  0.581  0.677  0.516  0.740   0.874  0.580  0.699  0.608  0.652  0.664  0.548   0.606  0.878  0.588  0.556  0.602  0.682   0.577  0.556 | 0.001  0.001  <0.001  <0.001  0.005  0.001  0.001  <0.001  0.003  <0.001  <0.001  0.001  <0.001  <0.001  <0.001  <0.001  0.002  <0.001  <0.001  0.001  0.001  <0.001  <0,001  0.001  0.001 |
| CD8 | CD45  CD45RO  Bcl2  CD14  CD20  CD27  CD3  CD34  CD4  CD44  CD56  CD86  FAPa  HLADR  PR  VISTA | 0.627  0.603  0.886  0.554  0.674  0.693  0.813   0.610  0.652  0.532  0.562   -0.503  0.612  0.612  0.732  0.696 | <0.001  <0.001  <0.001  0.001  <0.001  <0.001  <0.001  <0.001  <0.001  0.002  0.001  0.005  <0.001  <0.001  <0.001  <0.001 |
| CD34 | Bcl2  CD56  CD8  CD86  PTEN  VISTA | 0.721   0.570  0.610  -0.576  0.512  0.732 | <0.001  0.001  <0.001  0.001  0.004  <0.001 |
| HLA DR | CD45RO  Bcl2  CD11c  CD3  CD4  CD8 | 0.584  0.557  0.642  0.586  0.606  0.612 | 0.001  0.001  <0.001  0.001  <0.001  <0.001 |
| Tumor ROI’s of the short-term survivors | | | |
| PanCK | B4-1BB  EpCam | -0.539  0.641 | 0.008  0.001 |
| B4-1BB | PanCK  CD14  CD20  CD25  CD27  CD40  CD44  CD80  GITR  ICOS  IDO1 | -0.539  0.511  0.505  0.626  0.599  0.568  0.570  0.824  0.664  0.663  0.644 | 0.008  0.013  0.014  0.001  0.003  0.005  0.005  <0.001  0.001  0.001  0.001 |
| B7H3 | FAPa | 0.552 | 0.006 |
| Beta2 Microglobulin | NYESCO1CTag1B  CD127  StingTmem173 | 0.585  0.610  0.588 | 0.003  0.002  0.003 |
| CD127 | Beta2 Microglublin  CD25  CD44  PDL2  StingTmem173 | 0.610.  0.606  0.611  0.527  0.627 | 0.002  0.002  0.002  0.010  0.001) |
| CD25 | CD45RO  B4-1BB  CD11c  CD127  CD14  CD40  CD44  CD80  EpCam  FAPa  ICOS  PDL1 | 0.542  0.626   0.529  0.606  0.691  0.702  0.673  0.615  -0.553   0.646  0.588  0.516 | 0.008  0.001  0.010  0.002  <0.001  <0.001  <0.001  0.002  0.006  0.001  0.003  0.012 |
| CD66b | CD80  FoxP3  IDO1  LiverArginase | 0.560  0.540  0.512  0.893 | 0.005  0.008  0.013  <0.001 |
| CD80 | B4-1BB  CD14  CD25  CD40   CD44  CD66b  ICOS  IDO1 | 0.824  0.540  0.615  0.664  0.819  0.560  0.667   0.524 | <0.001  0.008  0.002  0.001  <0.001  0.005  0.001  0.010 |
| CTLA4 | αSMA  OX40L | 0.875  0.618 | <0.001  0.002 |
| FAPa | B7H3  CD14  CD25  CD44  CD86  Fibronectin  KI67 | 0.552  0.567  0.646  0.551  -0.614  0.621   0.512 | 0.006  0.005  0.001  0.006  0.002  0.002  0.013 |
| FoxP3 | CD66b  Fibronectin  LiverArginas | 0.540  -0.558  0.544 | 0.008  0.006  0.007 |
| GITR | B4-1BB  LAG3  PDL2 | 0.644  0.509  0.692 | 0.001  0.013  0.001 |
| HLA DR | CD45RO  Bcl  CD11c  CD14  CD163  CD20  CD27  CD3  CD4  CD4  CD8  ICOS  S100b  VISTA | 0.844  0.578  0.808  0.763  0.656  0.727  0.554  0.772  0.741  0.751  0.610  0.557  0.526  0.673 | <0.001  0.004  <0.001  <0.001  0.001  <0.001  0.006  <0.001  <0.001  <0.001  0.002  0.006  0.010  0.001 |
| KI67 | FAPa | 0.512 | 0.013 |
| LAG3 | ERalpha  GITR | 0.555  0.509 | 0.006  0.013 |
| LiverArginase | CD66b  FoxP3 | 0.893  0.544 | <0.001  0.007 |
| OX40L | CTLA4 | 0.618 | 0.002 |
| PD1 | CD11c  CD68  CD8  TIM3 | 0.540  0.587  0.524  0.650 | 0.008  0.003  0.010  0.001 |
| PDL1 | CD45RO  CD11c  CD20  CD25  CD40  CD8  MART1MelanA | 0.542  0.549  0.520  0.516  0.593  0.560  0.554 | 0.007  0.005  0.011  0.012  0.003  0.005  0.006 |
|  |  |  |  |

**Supplementary Table 7.** DSP results of the correlation analysis in the stromal ROIs.

| Cell type | Correlated cell type | Correlation coefficient (r) | p-value |
| --- | --- | --- | --- |
| Stroma like ROI’s of the long-term survivors | | | |
| CD14 | CD163 | 0.547 | 0.001 |
| CD68 | CD11c | 0.584 | 0.001 |
| CD8 | αSMA  CD45RO  B7H3  CD3  CD44  Fibronectin  HLA DR  PTEN | -0.734   0.577  -0.572  0.846   0.507  -0.644  0.515  -0.555 | 0.001  <0.001  0.001  0.001  0.002  <0.001  0.002  0.001 |
| ERalpha | Fibronectin  PTEN | 0.554  0.505 | 0.001  0.002 |
| Stroma like ROI’s of the short-term survivors | | | |
| aSMA | CD45RO  B7H3  Bcl  Beta2Microglublin  CD20  CD3  CD4  CD40  CD8  CTLA4  FAPa  FoxP3  HLA DR  IDO1  MART1MelanA  NYESCO1CTag1B  OX40L  PDL1  PDL2 | -0.660  0.715  -0.793  -0.521  - 0.749  -0.664  -0.543  -0.791  -0.505  0.890  0.696  0.506  -0.718  -0.549  0.551  0.557  0.846  0.684  -0.594 | 0.001  <0.001  <0.001  0.011  <0.001  0.001  0.007  <0.001  0.014  0.001  <0.001  0.014  <0.001  0.007  0.006  0.001  <0.001  p<0.001  0.003 |
| B4-1BB | CD34  CD8  CD86  EpCam  TIM3 | -0.561  -0.568  -0.651  0.619  -0.652 | 0.005  0.005  0.001  0.002  0.001 |
| Beta2microglublin | αSMA  CD45RO  CD11c  CD14  CD20  CD40  CD44  Fibronectin   FoxP3  HLADR  KI67  MART1MelanA  OX40L | -0.521  0.630  0.642  0.635  0.529  0.676  0.560  -0.641  -0.655  0.532  0.558  -0.501  -0.645 | 0.011  0.001  0.001  0.001  0.010  <0.001  0.005  0.001  0.001  0.009  0.006  0.015  0.001 |
| CD127 | CD3  CD4  S100b | -0.508  -0.570  -0.639 | 0.013  0.005  0.001 |
| CTLA4 | αSMA  CD45RO  B7H3  Bcl  CD20  CD3  CD4  CD40  CD8   FAPa  Fibronectin  HLA DR  IDO1  LAG3  OX40L  PDL2 | 0.890  -0.591  0.521  -0.875  -0.586  -0.696  -0.675  -0.691  -0.613  0.799  0.614  -0.751   -0.516  0.501  0.683  -0.621 | 0.001  0.003  0.011  p<0.001  0.003  0.001  <0.001  <0.001  0.002  <0.001  0.002  <0.001  0.012  0.015  <0.001  0.002 |
| FAPa | αSMA  B7H3  Bcl2  CTLA4  Fibronectin  HLADR  OX40L  PDL2 | 0.696  0.672  -0.877  0.799  0.514  -0.563  0.545  -0.675 | <0.001  <0.001  <0.001  <0.001  0.012  0.002  0.007  <0.001 |
| FoxP3 | αSMA  CD45RO  Beta2Microglublin  CD11c  CD14  CD34  CD40  OX40L | 0.506   -0.502  -0.655  -0.603  -0.562  -0.539  -0.540  0.540 | 0.014  0.015  0.001  0.002  0.002  0.008  0.008  0.008 |
| KI67 | Beta2Microglobulin  CD11c  PTEN | 0.558  0.559  -0.504 | 0.006  0.006  0.014 |
| OX40L | αSMA  CD45RO  B7H3  Bcl  Beta2Microgloblin  CD20  CD40  CTLA4  FAPa  Fibronectin  FoxP3  HLA DR   IDO1  PD1  VISTA | 0.846  -0.584  0.691  -0.570  -0.645  -0.654  -0.785  0.683  0.545  0.585  0.540   -0.609  -0.587  0.566  -0.518 | <0.001  0.003  <0.001  0.005  0.001  0.001  <0.001  <0.001  0.007  0.003  0.008  0.002  0.003  0.016  0.011 |
| PD1 | CD11c  CD8  TIM3 | -0.522  -0.528   -0.506 | 0.011  0.010  0.014 |
| PDL1 | αSMA  B7H3  Fibronectin  MART1MelanA | 0.684  0.626  -0.561  0.598 | <0.001  0.001  0.005  0.003 |
| StingTmem173 | GITR  VISTA | 0.521  0.659 | 0.011  0.001 |
| VISTA | CD14  GITR  OX40L  StingTmem173 | 0.586  0.558  -0.518  0.659 | 0.003  0.006  0.011  0.001 |
